# The stabilizing effect of an oligomeric proanthocyanidin on red blood cell membrane structure of poorly controlled Type II diabetes

**DOI:** 10.1038/nutd.2017.25

**Published:** 2017-05-15

**Authors:** J Visser, P J van Staden, P Soma, A V Buys, E Pretorius

**Affiliations:** 1 Department of Physiology, Faculty of Health Sciences, University of Pretoria, Arcadia, South Africa; 2 Department of Statistics, Faculty of Natural and Agricultural Sciences, University of Pretoria, Hatfield, South Africa; 3 Unit for Microscopy and Microanalysis, University of Pretoria, Arcadia, South Africa; 4 Department of Physiological Sciences, Stellenbosch University, Stellenbosch, South Africa

## Abstract

Type II diabetes (T2D) is a pandemic characterized by pathological circulating inflammatory markers, high-glucose levels and oxidative stress. The hematological system is especially vulnerable to these aberrant circulating molecules, and erythrocytes (RBCs) show aberrant rheology properties, owing to the direct contact with these molecules. Pathological levels of circulating inflammatory markers in T2D therefore have a direct effect on the molecular and cellular structure of RBCs. Previous research has suggested that antioxidants may reduce oxidative stress that results from the pathological inflammatory markers. Particularly, polyphenol antioxidants like oligomeric proanthocyanidins (OPCs) may act as a hydroxyl mopping agent, and may have a positive effect on the deformability and membrane protein structure of RBCs from T2D. In this paper, we look at the effect of one such agent, *Pinus massoniana* bark extract (standardized to 95% oligomeric proanthicyanidins), on the RBC membrane structures and RBC shape changes of T2D, after laboratory exposure at physiological levels. Our methods of choice were atomic force microscopy and scanning electron microscopy to study RBC elasticity and ultrastructure. Results showed that in our hands, this OPC could change both the eryptotic nature of the RBCs, as viewed with scanning electron microscopy, as well as the elasticity. We found a significant difference in variation between the elasticity measurement values between the RBCs before and after OPC exposure (*P*-value <0.0001). In conclusion, the data from both these techniques therefore suggest that OPC usage might contribute to the improvement of RBC functioning.

## Introduction

Type II diabetes (T2D) is commonly associated with comorbidities including dyslipidaemia^1, 2, 3, 4^ and hypertension.^4, 5^ The main cause of morbidity and mortality in individuals with T2D is cardiovascular disease (CVD), with up to 80% of diabetes subjects dying as a result of cardiovascular complications.^1, 3, 6^ The risk of atherothrombotic events is also comparable to individuals without diabetes but with a history of ischemic heart disease.^1^ Furthermore, the prognosis in these individuals following an event remains poor, despite major advances in treatment^1^ such as antiplatelet therapies, and the control of the modifiable risk factors like hypertension, obesity, smoking and dyslipidaemia.^7^

Changed inflammatory marker levels, oxidative stress and chronic (systemic) inflammation, all lead to a pathophysiological coagulation system that is characterized by hypercoagulability. Systemic inflammation has a fundamental role in many chronic conditions, including T2D and CVD.^8, 9, 10, 11, 12^ CVD and vascular dysfunction are fundamental parts of the etiology of T2D and this condition is also known to have a changed coagulation system. Therefore, all these factors serve as early indicators of vascular dysfunction. Thus, there are important links between oxidative stress, a changed inflammatory marker profile and inflammation, the progression of T2D and ultimately, vascular dysfunction.^4^

Dysregulated inflammatory markers, either upregulated or downregulated, are associated with diabetes.^4^ These inflammatory markers are mainly C-reactive protein,^13, 14^ coagulation factors,^14, 15, 16, 17, 18^ nitric oxide,^19, 20, 21, 22, 23^ tumor necrosis factor-α, nuclear factor kappa-B, cyclooxygenase-2,^24, 25, 26, 27, 28^ prostaglandin-2,^29, 30^ Interleukin-6^16, 17, 29, 31, 32^ and thromboxane A2.^33, 34^

A fundamental part of a pathological coagulation system and a systemic hypercoagulability, is an altered erythrocyte (RBC) function. All of the above-mentioned circulating inflammatory markers have a noticeable effect on RBC functioning, specifically on their rheology properties. Hypercoagulability affects fibrin (ogen) and this in turn also directly impacts on RBC rheology. It is also thought that abnormalities of high blood glucose as is the case in insulin resistance, and relative insulin deficiency, may have a negative effect on the structure and function of RBCs at molecular scale.^4^

Pathological levels of circulating inflammatory markers, high-glucose levels and the associated oxidative stress in T2D therefore have a direct effect on the molecular and cellular structure of RBCs. Their membranes consist of the typical lipid bilayer, the actin–spectrin cortex and the integral and peripheral membrane proteins,^35, 36^ of which ~20 are major proteins and at least 850 minor ones.^35^ In particular, flippases, floppases and scramblases (integral proteins) facilitate the transmembrane passage, as well as the structural arrangement of lipids RBC membranes. Structural alterations, owing to the systemic inflammatory profile in T2D, are known to affect the RBC membrane lipids, as well as band 3 and spectrin, which in turn cause RBC structural shape changes.^36, 37^ For an in-depth review of the RBC membrane structure, see reference Pretorius *et al.*^38^

AS oxidative stress is known to have a role in the progression of T2D^4, 23, 39, 40^ and biophysical shape changes of RBCs,^36, 41^ the question that now arose is whether there is a polyphenol antioxidant that may act as a hydroxyl mopping agent, and may have a positive effect on the deformability and membrane protein structure of RBCs from T2D that can be added to the diet. A group of (polyphenolic) antioxidants found in pine bark extract and grape seed extract that may have an effect, is the oligomeric proanthocyanidins (OPCs). Various preliminary clinical trials have been conducted investigating the effect of OPCs in several indications and clinical contexts including asthma, CVD and diabetes.^42, 43, 44, 45, 46, 47^

It has been shown that OPCs increase erythrocyte membrane fluidity *in vitro*, potentially due to inducing modification of the lipid bilayer and lipid–protein interactions as shown by Sivonova and co-workers in 2004.^42^ In an *in vitro* study, OPCs (0.07 mg ml^−1^) resulted in 50% inhibition of the formation of erythrocytes with elevated density (increased adherence to neutrophils, platelets and vascular endothelial cells) from patients with sickle cell anemia.^48^ OPCs have also been shown to have anti-inflammatory effects. It has been demonstrated to inhibited cyclooxygenase-1 and -2 activity^49^ and reducing the production of interleukin-1beta and its associated mRNA *in vitro*, as well as the expression of IL-2.^50^ Activation of two major transcription factors heavily involved in interleukin-1beta gene expression, nuclear factor kappa-B and activator protein-1, have also been shown to be reduced.

In the current study, we therefore investigated the effect of the addition of physiological levels of OPCs (*Pinus massoniana* bark extract standardized to 95% oligomeric proanthicyanidins), on the RBC membrane structures and RBC shape changes of T2D. Shape changes or patho-morphological changes are closely associated with RBC properties including area deformability, such as membrane elasticity changes due to membrane biochemical changes as discussed previously, and directly translate as biophysical indicators. In this study, atomic force microscopy (AFM) was used to evaluate membrane elasticity, whereas scanning electron microscopy (SEM) was used to evaluate physical changes. Although a randomized control trial is obviously the most useful way to establish the effect of any molecule (dietary or otherwise) it is of great importance to first establish if the molecule has any effect *in vitro*.

## Materials and methods

This study was approved by the Ethical Committee of the University of Pretoria (South Africa). A written form of informed consent was obtained from all healthy donors (available on request). The methods were carried out in accordance with the approved guidelines. Blood was collected and methods were carried out in accordance with the relevant guidelines of the ethics committee (ethics number: 506/2014 and 298/2016: E Pretorius and J Bester: principal investigators for use of control blood; ethics number: 151/2006 for T2D study). We adhered strictly to the Declaration of Helsinki.

### Samples used

We recruited 20 healthy individuals with no systemic inflammation, and who did not use any chronic medication, as our control group. T2D individuals were voluntarily recruited, from the Diabetic Clinic at the Steve Biko academic hospital, Pretoria, South Africa. Demographic data including age, gender, glucose level at time of sample collection, hemoglobin A1c (HbA1c) measures and medication used for typical comorbidities of T2D were recorded. Inclusion criteria consisted of both male and female participants, aged between 19 and 70, with a diagnosis of T2D for >3 months prior to screening and without any signs of infection. Smoking was an exclusion criteria and our samples consisted of individuals (control and T2D) who did not smoke. Diabetic individuals were diagnosed according to the Society for Endocrinology, Metabolism and Diabetes of South Africa (SEMSDA) guidelines. These guidelines follow the American Diabetes Association (ADA) criteria to define T2D.

An important consideration might be that these OPCs should not interact negatively with the currently used medications that typical poorly controlled diabetes patients may be taking. Therefore, a literature search was also done to investigate possible interactions of OPCs with medication typically prescribed for comorbidities associated with T2D.

### Dosage of OPCs

The standard daily dosage of pine bark extract standardized to 95% OPCs for people with diabetes is 50–200 mg per day.^43, 47, 51, 52^ The extract that was used is a 4:1 concentrated extract. Therefore, in order to reach a daily dosage of 200 mg, 50 mg was used. Based on an average individual of 70 kg, the final concentration in the blood is expected to be 9 μg ml^−1^. Therefore, the exposure concentration for both the AFM and SEM experiments was 9 μg ml^−1^.

### Sample preparation for AFM

Whole blood samples were centrifuged at 145 g for 30 s. The supernatant consisting of plasma, platelets and leukocytes, was discarded. The remaining RBCs were prepared for AFM by fixing in 4% formaldehyde made up in phosphate-buffered saline for 30 min, at room temperature (22 °C) followed by dehydration. RBCs were suspended in undiluted (161.39 g mol^−1^) hexamethyldisilazane onto a glass cover slip by placing a drop and spreading the fluid by tilting the cover slip sideways, ensuring an even distribution of cells. The cover slips were then dried and stored until AFM analysis.

### AFM imaging and measurement

The cells were characterized with a commercial AFM system (Dimension Icon with ScanAsyst, Bruker, Santa Barbara, CA, USA) using the PeakForce QNM (Quantitative Nanomechanical Property Mapping) imaging mode. This method is similar to the standard tapping mode of scanning probe microscopy, where the probe and the sample are brought together intermittently. See Bester and co-workers 2013,^53^ for a comprehensive discussion of the AFM methods used in this paper. This mode operates by controlling the maximum force applied by the probe to the sample^54^ and allows that at every pixel a rapid force–distance curve is generated. The cantilever’s deflection sensitivity and spring constant is calibrated before measurements, therefore the curve can be analyzed quantitatively to obtain a series of specific property maps of the sample. The retract curve can then be used to calculate the slope of the curve (which is called the modulus) and the minimum of the curve (otherwise known as adhesion images). The deformation can therefore be calculated and this is the variation between the zero and maximum force. Following this, the area between the approach and retract curve can then be used to calculate energy dissipation.^53, 55, 56^ The stiffness of an elastic material can be measured using Young’s modulus.^36, 57, 58^ Young’s modulus is the stress divided by the corresponding strain, with greater values indicating increased stiffness or decreased deformability. As each force curve’s data, can also be stored individually, and quantitative measurements of the Young’s modulus can be generated by fitting the slope of any force–distance curve of the image to an appropriate model (in this instance; the Derjaguin–Muller–Toporov Model.^59^

Silicon Nitride probes (TAP525−MPP 13120−10, Bruker, USA) were employed in all AFM measurements and these probes were specified to have a nominal force constant of 200 N.m-1, a resonant frequency between 430 and 516 kHz (measured by the manufacturer), and a nominal tip radius of 15 nm. Ten cells per participant were analyzed by selecting a 1μm by 1μm scan area on the periphery of the RBC and performing 128 by 128 data points of individual force curve measurements with a peak force of 6 μN. As there might be differences in concavity of RBCs, the periphery of the cells was chosen to ensure that an area that is not affected by the concavity of the specific RBC is measured. The scans were performed at 0.6 Hz, which is a tip velocity of 1.2 μm s^−1^ and 50 force curves were chosen at random within the stated area. The offline software (NanoScope Analysis version R3, Bruker, USA) was used to process the force curves. A goodness-of-fit model was used to obtain the coefficient of determination (*R*^2^) between the modulus model and the data given by the acquired curve, determined by calculating the ratio of explained variation to total variation in the data set. We only used force curves with a coefficient of determination of 0.85 and above for modulus measurements.

### Atomic force microscopy

To have statistical power of 95% to detect a statistically significant treatment effect at a 1% significance level, a sample of 35 T2D patients is required, whereas 47 patients are needed for statistical power of 99% (see Rosner^60^) for details on sample size calculations in before–after studies). A sample of 60 T2D patients was recruited and AFM data (RBC membrane elasticity measurements) were available for 56 of these individuals. The elasticity measurement distributions were analyzed for each patient. Owing to the asymmetry of these distributions, it was decided to calculate for each patient the median elasticity measurement value from the corresponding nave T2D blood sample. The same was done for each patient for the T2D blood sample exposed to OPCs. Thus, we obtained 56 matched (paired) median values to be utilized as reference values in the various graphical and statistical analyses done. These analyses included the drawing of histograms and box and whisker diagrams, testing for normality, testing for a difference in the medians between the nave T2D blood samples and T2D blood samples exposed to OPCs and testing for a difference in the variation (spread) between these two groups of blood samples.

Statistical analyses were done using SAS/STAT and SAS/IML software, Version 9.4 of the SAS System for Windows (SAS Institute, Cary, NC, USA). The histograms and box and whisker diagrams were drawn with Wolfram Mathematica, Version 10.4 (Wolfram Research, Champaign, IL, USA).

### Scanning electron microscopy

The SEM experiment was conducted by comparing the naive T2D blood samples with T2D blood samples exposed to OPCs. Whole blood samples from the 20 healthy individuals were also collected, scanned and analyzed as a control group. After the blood was collected, 10 μl of whole blood with 5 μl thrombin (to create an extensive fibrin network) were placed directly on a glass cover slip, fixed, dehydrated, dried, mounted and coated with carbon according to previously described methods.^4, 61^ A Zeiss ULTRA Plus FEG-SEM with InLens capabilities was used and micrographs were taken at 1 kV.

## Results

The demographic data for the group of 60 patients with T2D is summarized in Table 1. The information collected included glucose measured at the time of sample collection, HbA1c values as well as chronic medication typically administered to T2D patients. Table 1 shows that, although almost all T2D participants (97%) are taking hypoglycaemic medication, the samples group still represents a poorly controlled diabetic population. Besides medication for glucose control, the T2D group also includes individuals on medication for dyslipidaemia and hypertension, whereas some are taking anticoagulants. The control group of 20 healthy individuals was selected to be age and gender matched to the T2D group.

**Figure 1:**
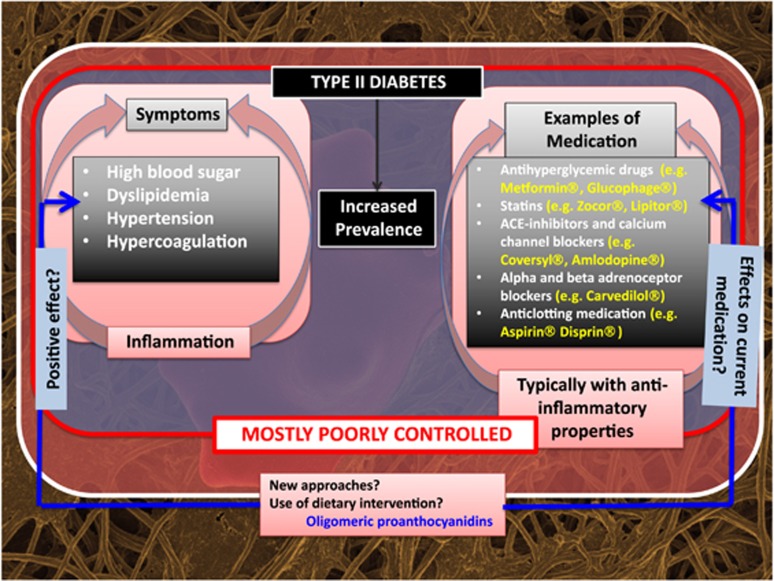
Type II diabetes, symptoms suggestive of systemic inflammation, typical medication for the comorbidities and solution for disease tracking based on an individualized, precision medicine approach.

An important consideration for this study was the possible interactions of OPCs with the currently used mediations that typical poorly controlled diabetes patients may be taking (see Figure 1). Table 2 shows possible interactions of OPCs with typically prescribed chronic medication.

**Figure 2:**
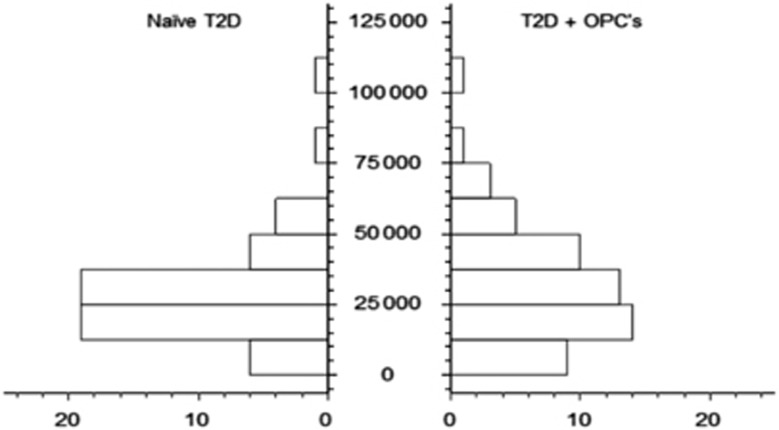
Paired histograms comparing the group of participants with type II diabetes (naive T2D) and the same group of participants treated with OPCs (T2D+OPCs).

**Figure 3:**
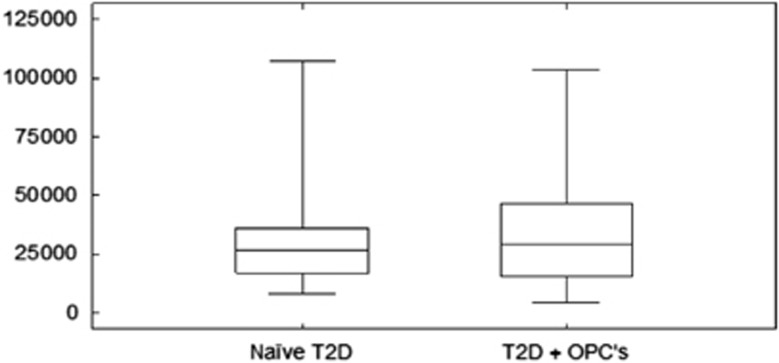
Box and whisker diagrams for the group of participants with type II diabetes (naive T2D) and the same group of participants treated with OPCs (T2D+OPCs).

**Figure 4:**
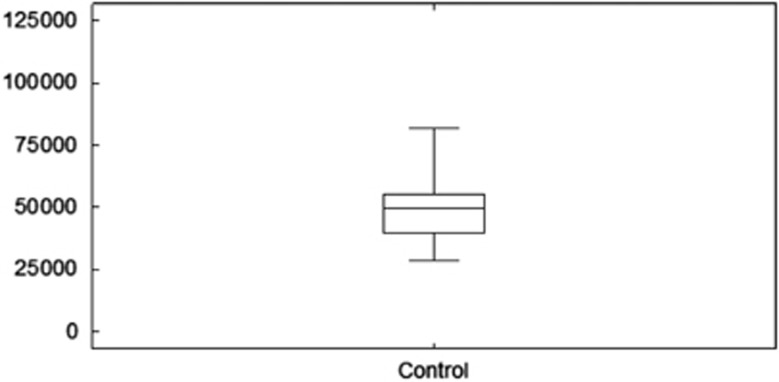
Box and whisker diagram for the control group of 20 healthy individuals.

Descriptive statistics for the naive T2D group and the T2D group treated with OPCs are summarized in Table 3, whereas Figures 2 and 3 illustrate paired histograms and box and whisker diagrams for the two diabetes groups. It is clear from these diagrams that both groups are positively skewed and hence non-normal. The non-normality was confirmed with various tests for normality, including the Anderson–Darling^62^ and Shapiro–Wilk tests^63^ (see Table 3). Figure 4 shows a box and whisker diagram for the control group of 20 healthy individuals.

Owing to the non-normality of the data, non-parametric hypothesis tests were utilized to compare the elasticity measurement values between the two groups of blood samples. First, with the Wilcoxon matched pairs test^64^ no statistically significant difference in the median elasticity measurement values between the naive T2D blood samples and T2D blood samples exposed to OPCs was found (*P*-value=0.3083). This is also evident from the box and whisker diagrams in Figure 3. However, these box and whisker diagrams do suggest that the variation in elasticity measurement values for the T2D group treated with OPCs is larger than the variation in elasticity measurement values for the naive T2D group. Using the modification of Wilcox^65^ of the Morgan–Pitman test,^66, 67^ a significant difference in variation between the elasticity measurement values of the two groups of blood samples was found (*P*-value<0.0001).

### Scanning electron microscopy

**Figure 5:**
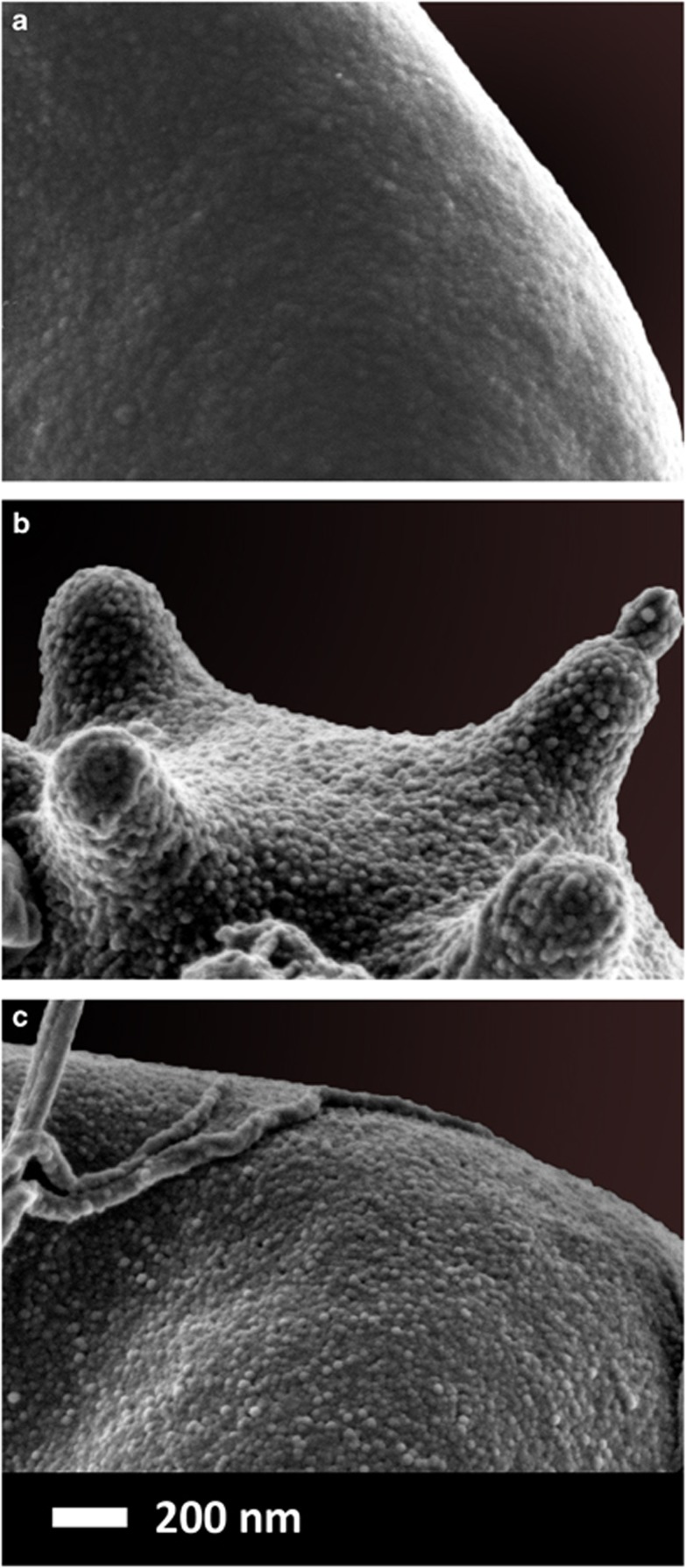
(**a**) A typical RBC membrane from a healthy individual. (**b**) A typical RBC membrane from a type II diabetes individual. (**c**) A typical RBC membrane from a type II diabetes individual, treated with an OPC.

SEM followed the same trends as the AFM results, and it seems as if the OPC changes the RBC membrane to be comparable to that of a typical healthy RBC membrane (see Figure 5). A healthy RBC membrane shows a smooth undulating structure, where RBCs from T2D are known to be eryptotic, with a membrane that is much rougher.^4, 61^

Eryptosis is programmed RBC cell death, similar to that of apoptosis, and is found in (all) inflammatory conditions. For comprehensive reviews on this process, see Pretorius *et al.*^38^ With the addition of the OPC, the eryptotic RBC structure is reversed, and the membrane roughness are slightly improved, compared with that of a typical healthy RBC.

## Discussion

In a healthy RBC, the neutral phosphatidylcholine and sphingomyelin are mostly found on the outside, and the charged phosphatidylserine, phosphatidylinositol and phosphatidylethanolamine, are found mostly on the inner membrane leaflet.^36, 41^ In most inflammatory conditions, RBCs have been noted to show eryptosis (similar to apoptosis, but called eryptosis owing to theabsence of mitochondria and nuclei); for extensive reviews on eryptosis see various publications.^38, 68, 69, 70, 71, 72, 73^ During eryptosis, a membrane phosphatidylserine-flip occurs, and phosphatidylserine is then present on the outside leaflet of the RBC membranes.^74, 75, 76, 77, 78, 79^ Ultimately, eryptosis results in an inflammatory RBC presence in the circulation. Eryptotic RBCs are known to themselves produce membrane microparticles and these microparticles in itself are inflammatory.^80^ In all inflammatory conditions, RBCs demonstrate enhanced eryptosis and a pathological membrane structure with a changed elasticity. This was previously also demonstrated in T2D.^61, 81^ The pathological nature T2D RBCs, together with the general hypercoagulability seen in the condition,^82^ fundamentally influences the inflammatory profiles of the patients. Eryptosis is also seen in conditions like anemia.^83^ Previously, it was also shown that pathological RBC structure, with an accompanying changed viscoelasticity and hypercoagulability of whole blood and plasma, are found in conditions like Alzheimer’s and Parkinson’s disease,^84, 85, 86^ hyperferritinemia and haemochromatosis.^87^

Procyanidins and proanthocyanidins have been shown to have antioxidative and anti-inflammatory properties.^88, 89^ Preliminary clinical trials have been conducted in a number of indications and clinical contexts, including asthma, CVD and diabetes.^42, 43, 44, 45, 46, 47, 51, 90, 91^ It has been shown that OPCs increase RBC membrane fluidity *in vitro*, potentially owing to inducing modification of the lipid bilayer and lipid–protein interactions as shown by Sivonová *et al.*^42^ Sivonová *et al.* studied the *in vitro* effect of polyphenol rich plant extract, trademarked as Pycnogenol, on RBC membrane fluidity. They found that the extract significantly increased the membrane fluidity especially at the membrane surface as compared with untreated RBCs. They also found that the extract had protective effect against lipid peroxidation, generation of thiobarbituric acid reactive products and oxidative hemolysis induced by H_2_O_2_. They stated that, even though the exact mechanisms are unknown, it is thought that pycnogenol can reduce the lipid peroxidation and oxidative hemolysis either by reducing free radicals or by chelating metal ions, or by both. They concluded that the extract possibly modifies membrane-dependent processes not only by its chemical action, but also by interacting directly with cell membranes and/or penetrating the membrane and therefore inducing modification of the lipid bilayer and lipid–protein interactions.^42^

In the current study, the protective antioxidant effect of OPCs on the local mechanical properties of the erythrocyte membrane was evaluated, and therefore the membrane elasticity of erythrocytes of patients with T2D, using AFM techniques.

The Wilcoxon matched pairs test^64^ showed no statistically significant difference in the median elasticity measurement values between the T2D sample group and the same group exposed to OPCs (*P*-value=0.3083). From this data it can be interpreted that the OPCs had no effect, or at least not a measurable effect, on the local mechanical properties of the erythrocyte membrane by means of AFM, and therefore the membrane elasticity of erythrocytes.

Using the modification of Wilcox^65^ of the Morgan–Pitman test,^66, 67^ a significant difference in variation between the elasticity measurement values of the two groups was found (*P*-value <0.0001). Oxidative stress has been associated with premature erythrocyte aging.^92^ It can therefore be assumed that in T2D more erythrocytes would be expected to measure as aged, lesser deformable erythrocytes^92^ as seen in this study and to have smaller Young’s modulus values and a smaller variance in membrane elasticity as seen by the AFM data (refer to Figure 3). The results showed that at least some of the samples were positively affected in the treated T2D group. It can therefore be presumed that the protective antioxidant effect of OPCs on the local mechanical properties of the erythrocyte membrane, and therefore the membrane elasticity of patients with T2D as measured by AFM, has an improving effect in comparison with the untreated T2D group.

These results are comparable to the findings of Pretorius (2015) where the authors investigated the effect of iron chelators deferoxamine or deferasirox on T2D erythrocytes by means of SEM and AFM. They found that treatment with either deferasirox or deferoxamine improved the Young’s modulus values towards normal values. This indicates a possible improvement in the elasticity of the cells.^4^ However, when they compared the elasticity measurements individually after treatment, a more complex picture emerges owing to the substantial variation in the two groups.^4^ Similar variation was found in this study. In this study we found that a detailed analysis showed that the effect of the iron chelators on membrane elasticity was patient-specific.^4^

This is further supported after closer inspection, at × 150 000 magnification (refer to Figure 5), the erythrocyte membranes are visible. The T2D membrane ultrastructure appears much more rough and textured than that of healthy erythrocytes, possibly due to membrane phospholipid scrambling, associated with oxidative stress.^86^ The OPCs-treated sample shows a visibly smoother surface of the erythrocyte membranes, as compared with the untreated T2D erythrocytes.

It is also important to note that AFM is generally considered to be an informative technique that is frequently used in erythrocyte structural studies using Young’s modulus to measure membrane stiffness and elasticity.^36, 57, 58^ However, in this study relatively complex statistical methodology was needed to statistically compare treated and untreated T2D groups. The technique was also found to be a timeous technique when a larger samples group is being studied. In addition, it is considered to be a complicated method to implement as a patient-orientated approach.^36^

## Conclusion

In conclusion, the protective antioxidant properties of OPCs improved membrane elasticity as shown by a significant difference (*P*-value<0.0001) in variation between the elasticity measurement values of the two groups T2D treated and untreated) when using the modification of Wilcox^65^ of the Morgan–Pitman test.^66, 67^ Using SEM analysis these findings are further supported by the visibly smoother membrane surface viewed at × 150 000 magnification. The data from AFM showed that this is possibly due to an improvement towards a more normal variance of elasticity, and possibly contributing to the improvement of biophysical shape changes as seen in SEM analyses.

## Figures and Tables

**Table 1:** Summary of patient demographic data and medication

*TYPE II* *diabetes demographic data and medication summary*	
*% Distributions*	*Individuals with diabetes*
Number of female patients (%)	34 (57%)
Number of male patients (%)	26 (43%)
Total no of patients	60
Average age±s.d.	56.7±9.9
Average HbA1c (%)±s.d.	9.0±2.2
Average glucose level (mmol l^−1^)±s.d.	10.1±5.1
Number of patients taking anticholesterol medication (%)	40 (67%)
Number of patients taking hypoglycaemic medication (%)	58 (97%)
Number of patients taking anticoagulants medication (%)	31 (52%)
Number of patients taking antihypertensive medication (%)	44 (73%)

**Table 2:** Medication typically administered to diabetes type II patients and possible interactions with OPCs

*Medication*	*Selected references*
*Glucose control*	
Effect on blood glucose levels in general	No evidence of interaction
Antihyperglycemic drug dimethylbiguanide (metformin/glucophage)	No evidence of interaction
Actraphane (mixture of fast-acting insulin and long-acting insulin)	No evidence of interaction
Actrapid (human soluble insulin)	No evidence of interaction
Humulin (70% human insulin isophane suspension and 30% human insulin injection (rDNA origin))	No evidence of interaction
Protophane (intermediate-acting insulin)	No evidence of interaction
	
*Hypertension control*	
Effect on blood pressure in general	Had no effect on blood pressure or heart rate.^46, 55^ In patients with mild blood pressure, blood pressure was normalized and thromboxane levels lowered.^47, 56^
Coversyl (active ingredient is perindopril arginine which is a angiotensin-converting enzyme inhibitor)	No evidence of interaction
Amlodopine (calcium channel blockers)	Reduced the dosage of the calcium channel blocker nifedipine significantly; plasma levels of endothelin-1 were reduced and concentrations of prostacyclin were elevated^47, 57^
Carvedilol (beta and alpha adrenoceptor blocker with antioxidant activity)	No evidence of interaction
Adalat (nifedipine) calcium channel blocker)	No evidence of interaction
	
*Anti-clotting medication*	
Aspirin (acetylsalicylic acid)	An inhibitory effect on platelet aggregation similar to aspirin.^44, 47, 55, 56, 58^ Does not affect INR (bleeding tendency) in patients taking aspirin.^46^
	
*Cholesterol medication*	
Effect on blood lipid levels in general	Decreases LDL-cholesterol, increases HDL-cholesterol levels, no significant change in total cholesterol or triglycerides.^46, 59^ No statistically significant improvement in total cholesterol or LDL levels compared with placebo.^44, 60^
Simvastatin	No evidence of interaction
Lipitor	No evidence of interaction

**Table 3:** Descriptive statistics and normality tests for the group of participants with type II diabetes (naive T2D) and for the same group of participants treated with OPCs (T2D+OPCs)

	*Naive T2D*	*T2D group treated with OPCS*
*Descriptive statistics*		
Sample size	56	56
Mean	30197.76	32730.20
s.d.	17693.60	21559.00
Median	26622.00	29326.25
75 percentile	36202.50	46812.00
25 percentile	16952.00	15545.80
Interquartile range	19250.50	31266.20
Maximum	106986.50	103698.00
Minimum	8093.00	4499.00
Range	98893.50	99199.00
		
P-*values for normality tests*		
Anderson–Darling	0.0001	0.0270
Shapiro–Wilk	<0.0001	0.0047

Abbreviations: OPCs; oligomeric proanthocyanidins; T2D, type II diabetes.

## References

[bib1] Alzahrani SH, Ajjan RA. Coagulation and fibrinolysis in diabetes. Diab Vasc Dis Res 2010; 7: 260–273. 20847109 10.1177/1479164110383723

[bib2] Klop B, Elte JWF, Cabezas MC. Dyslipidemia in obesity: mechanisms and potential targets. Nutrients 2013; 5: 1218–1240. 23584084 10.3390/nu5041218 PMC3705344

[bib3] Lorber D. Importance of cardiovascular disease risk management in patients with type 2 diabetes mellitus. Diabetes Metab Syndr Obes 2014; 7: 169–183. 24920930 10.2147/DMSO.S61438 PMC4043722

[bib4] Pretorius E, Bester J, Vermeulen N, Alummoottil S, Soma P, Buys AV et al. Poorly controlled type 2 diabetes is accompanied by significant morphological and ultrastructural changes in both erythrocytes and in thrombin-generated fibrin: implications for diagnostics. Cardiovasc Diabetol 2015; 14: 30. 25848817 10.1186/s12933-015-0192-5 PMC4364097

[bib5] Meusel L-AC, Kansal N, Tchistiakova E, Yuen W, MacIntosh BJ, Greenwood CE et al. A systematic review of type 2 diabetes mellitus and hypertension in imaging studies of cognitive aging: time to establish new norms. Front Aging Neurosci 2014; 6: 148. 25071557 10.3389/fnagi.2014.00148 PMC4085499

[bib6] Vazzana N, Ranalli P, Cuccurullo C, Davì G. Diabetes mellitus and thrombosis. Thromb Res 2012; 129: 371–377. 22197180 10.1016/j.thromres.2011.11.052

[bib7] Soma P, Pretorius E. Interplay between ultrastructural findings and atherothrombotic complications in type 2 diabetes mellitus. Cardiovasc Diabetol 2015; 14: 96. 26228646 10.1186/s12933-015-0261-9 PMC4521497

[bib8] Ruiz-Canela M, Bes-Rastrollo M, Martínez-González MA. The role of dietary inflammatory index in cardiovascular disease, metabolic syndrome and mortality. Int J Mol Sci 2016; 17: E1265. 27527152 10.3390/ijms17081265 PMC5000663

[bib9] Ross R. Atherosclerosis–an inflammatory disease. N Engl J Med 1999; 340: 115–126. 9887164 10.1056/NEJM199901143400207

[bib10] Frostegard J. Immunity, atherosclerosis and cardiovascular disease. BMC Med 2013; 11: 117. 23635324 10.1186/1741-7015-11-117 PMC3658954

[bib11] Gregor MF, Hotamisligil GS. Inflammatory mechanisms in obesity. Annu Rev Immunol 2011; 29: 415–445. 21219177 10.1146/annurev-immunol-031210-101322

[bib12] Hansson GK. Inflammation, atherosclerosis, and coronary artery disease. N Engl J Med 2005; 352: 1685–1695. 15843671 10.1056/NEJMra043430

[bib13] Kitada M, Zhang Z, Mima A, King GL. Molecular mechanisms of diabetic vascular complications. J Diabetes Investig 2010; 1: 77–89. 10.1111/j.2040-1124.2010.00018.x PMC4008020 24843412

[bib14] Kim HK, Kim JE, Park SH, Kim YI, Nam-Goong IS, Kim ES. High coagulation factor levels and low protein C levels contribute to enhanced thrombin generation in patients with diabetes who do not have macrovascular complications. J Diabetes Complications 2014; 28: 365–369. 24560421 10.1016/j.jdiacomp.2014.01.006

[bib15] Cucuianu M, Coca M. Thrombotic tendency in diabetes mellitus. Revisiting and revising a study initiated 30 years ago. Rom J Intern Med 2012; 50: 107–115. 23326954

[bib16] Goldberg RB. Cytokine and cytokine-like inflammation markers, endothelial dysfunction, and imbalanced coagulation in development of diabetes and its complications. J Clin Endocrinol Metab 2009; 94: 3171–3182. 19509100 10.1210/jc.2008-2534

[bib17] Tousoulis D, Papageorgiou N, Androulakis E, Siasos G, Latsios G, Tentolouris K et al. Diabetes mellitus-associated vascular impairment: novel circulating biomarkers and therapeutic approaches. J Am Coll Cardiol 2013; 62: 667–676. 23948511 10.1016/j.jacc.2013.03.089

[bib18] Madan R, Gupt B, Saluja S, Kansra UC, Tripathi BK, Guliani BP. Coagulation profile in diabetes and its association with diabetic microvascular complications. J Assoc Physicians India 2010; 58: 481–484. 21189694

[bib19] Khanna S, Singh GB, Khullar M. Nitric oxide synthases and diabetic cardiomyopathy. Nitric Oxide 2014; 43: 29–34. 25153033 10.1016/j.niox.2014.08.004

[bib20] Dellamea BS, Leitão CB, Friedman R, Canani LH. Nitric oxide system and diabetic nephropathy. Diabetol Metab Syndr 2014; 6: 17. 24520999 10.1186/1758-5996-6-17 PMC3928920

[bib21] Dzugkoev SG, Metel'skaya VA, Dzugkoeva FS. Effects of endogenous regulators of endothelial NO synthase on nitric oxide homeostasis and blood serum lipoproteins during experimental diabetes mellitus. Bull Exp Biol Med 2013; 156: 205–208. 24319749 10.1007/s10517-013-2311-1

[bib22] Schneider MP, Ott C, Schmidt S, Kistner I, Friedrich S, Schmieder RE. Poor glycemic control is related to increased nitric oxide activity within the renal circulation of patients with type 2 diabetes. Diabetes Care 2013; 36: 4071–4075. 24130344 10.2337/dc13-0806 PMC3836138

[bib23] Cohen RA, Tong X. Vascular oxidative stress: the common link in hypertensive and diabetic vascular disease. J Cardiovasc Pharmacol 2010; 55: 308–316. 20422735 10.1097/fjc.0b013e3181d89670 PMC3018745

[bib24] Kassan M, Choi SK, Galan M, Bishop A, Umezawa K, Trebak M et al. Enhanced NF-kappaB activity impairs vascular function through PARP-1-, SP-1-, and COX-2-dependent mechanisms in type 2 diabetes. Diabetes 2013; 62: 2078–2087. 23349490 10.2337/db12-1374 PMC3661639

[bib25] Wong WT, Tian XY, Huang Y. Endothelial dysfunction in diabetes and hypertension: cross talk in RAS, BMP4, and ROS-dependent COX-2-derived prostanoids. J Cardiovasc Pharmacol 2013; 61: 204–214. 23232839 10.1097/FJC.0b013e31827fe46e

[bib26] Rudock ME, Liu Y, Ziegler JT, Allen SG, Lehtinen AB, Freedman BI et al. Association of polymorphisms in cyclooxygenase (COX)-2 with coronary and carotid calcium in the Diabetes Heart Study. Atherosclerosis 2009; 203: 459–465. 18768181 10.1016/j.atherosclerosis.2008.07.018 PMC2699582

[bib27] Kellogg AP, Cheng HT, Pop-Busui R. Cyclooxygenase-2 pathway as a potential therapeutic target in diabetic peripheral neuropathy. Curr Drug Targets 2008; 9: 68–76. 18220714 10.2174/138945008783431691

[bib28] Bagi Z, Erdei N, Papp Z, Edes I, Koller A. Up-regulation of vascular cyclooxygenase-2 in diabetes mellitus. Pharmacol Rep 2006; 58(Suppl): 52–56. 17332672

[bib29] Schoenberger SD, Kim SJ, Sheng J, Rezaei KA, Lalezary M, Cherney E. Increased prostaglandin E2 (PGE2) levels in proliferative diabetic retinopathy, and correlation with VEGF and inflammatory cytokines. Invest Ophthalmol Vis Sci 2012; 53: 5906–5911. 22871833 10.1167/iovs.12-10410

[bib30] Salvi GE, Beck JD, Offenbacher S. PGE2, IL-1 beta, and TNF-alpha responses in diabetics as modifiers of periodontal disease expression. Ann Periodontol 1998; 3: 40–50. 9722689 10.1902/annals.1998.3.1.40

[bib31] Wang X, Bao W, Liu J, Ouyang YY, Wang D, Rong S et al. Inflammatory markers and risk of type 2 diabetes: a systematic review and meta-analysis. Diabetes Care 2013; 36: 166–175. 23264288 10.2337/dc12-0702 PMC3526249

[bib32] Kampoli AM, Tousoulis D, Briasoulis A, Latsios G, Papageorgiou N, Stefanadis C. Potential pathogenic inflammatory mechanisms of endothelial dysfunction induced by type 2 diabetes mellitus. Curr Pharm Des 2011; 17: 4147–4158. 22204375 10.2174/138161211798764825

[bib33] Zuccollo A, Shi C, Mastroianni R, Maitland-Toolan KA, Weisbrod RM, Zang M et al. The thromboxane A2 receptor antagonist S18886 prevents enhanced atherogenesis caused by diabetes mellitus. Circulation 2005; 112: 3001–3008. 16260636 10.1161/CIRCULATIONAHA.105.581892

[bib34] Winocour PD. Platelets, vascular disease, and diabetes mellitus. Can J Physiol Pharmacol 1994; 72: 295–303. 8069776 10.1139/y94-045

[bib35] Andolfo I, Russo R, Gambale A, Iolascon A. New insights on hereditary erythrocyte membrane defects. Haematologica 2016; 101: 1284–1294. 27756835 10.3324/haematol.2016.142463 PMC5394881

[bib36] Pretorius E, Olumuyiwa-Akeredolu OO, Mbotwe S, Bester J. Erythrocytes and their role as health indicator: Using structure in a patient-orientated precision medicine approach. Blood Rev 2016; 4: 263–274. 10.1016/j.blre.2016.01.001 26878812

[bib37] van Meer G. Dynamic transbilayer lipid asymmetry. Cold Spring Harb Perspect Biol 2011; 3: a004671. 21436058 10.1101/cshperspect.a004671 PMC3101844

[bib38] Pretorius E, du Plooy J, Bester J. A comprehensive review on eryptosis. Cell Physiol Biochem 2016; 39: 1977–2000. 27771701 10.1159/000447895

[bib39] Rees MD, Kennett EC, Whitelock JM, Davies MJ. Oxidative damage to extracellular matrix and its role in human pathologies. Free Radic Biol Med 2008; 44: 1973–2001. 18423414 10.1016/j.freeradbiomed.2008.03.016

[bib40] Leopold JA, Loscalzo J. Oxidative risk for atherothrombotic cardiovascular disease. Free Radic Biol Med 2009; 47: 1673–1706. 19751821 10.1016/j.freeradbiomed.2009.09.009 PMC2797369

[bib41] Arashiki N, Saito M, Koshino I, Kamata K, Hale J, Mohandas N et al. An unrecognized function of cholesterol: regulating the mechanism controlling membrane phospholipid asymmetry. Biochemistry 2016; 55: 3504–3513. 27267274 10.1021/acs.biochem.6b00407 PMC5288641

[bib42] Sivonová M, Waczulíková I, Kilanczyk E, Hrnciarová M, Bryszewska M, Klajnert B et al. The effect of Pycnogenol on the erythrocyte membrane fluidity. Gen Physiol Biophys 2004; 23: 39–51. 15270128

[bib43] Oliff H. Proprietary Botanical Ingredient Scientific and Clinical Monograph for Pycnogenol (French Maritime pine bark extract). American Botanical Council: Monograph. http://abc.herbalgram.org/site/DocServer/Pycnog_FullMono120809_LOW.pdf?docID=1741 2010.

[bib44] Preuss HG, Wallerstedt D, Talpur N, Tutuncuoglu SO, Echard B, Myers A et al. Effects of niacin-bound chromium and grape seed proanthocyanidin extract on the lipid profile of hypercholesterolemic subjects: a pilot study. J Med 2000; 31: 227–246. 11508317

[bib45] Cho K-J, Yun C-H, Yoon D-Y, Cho Y-S, Rimbach G, Packer L et al. Effect of bioflavonoids extracted from the bark of Pinus maritima on proinflammatory cytokine interleukin-1 production in lipopolysaccharide-stimulated RAW 264.7. Toxicol Appl Pharmacol 2000; 168: 64–71. 11000101 10.1006/taap.2000.9001

[bib46] Rohdewald P. A review of the French maritime pine bark extract (Pycnogenol), a herbal medication with a diverse clinical pharmacology. Int J. Clin Pharmacol Ther 2002; 40: 158–168. 10.5414/cpp40158 11996210

[bib47] Rohdewald PJ, Coates P, Blackman M, Cragg G, Levine M, Moss J et al Encyclopedia of Dietary Supplements. 2005, pp: 545-53.

[bib48] Ohnishi ST, Ohnishi T, Ogunmola GB. Sickle cell anemia: a potential nutritional approach for a molecular disease. Nutrition 2000; 16: 330–338. 10793299 10.1016/s0899-9007(00)00257-4

[bib49] Schäfer A, Chovanová Z, Muchová J, Sumegová K, Liptáková A, Ďuračková Z et al. Inhibition of COX-1 and COX-2 activity by plasma of human volunteers after ingestion of French maritime pine bark extract (Pycnogenol). Biomed Pharmacother 2006; 60: 5–9. 16330178 10.1016/j.biopha.2005.08.006

[bib50] Cho KJ, Yun CH, Packer L, Chunga AS. Inhibition mechanisms of bioflavonoids extracted from the bark of Pinus maritima on the expression of proinflammatory cytokines. Ann N Y Acad Sci 2001; 928: 141–156. 11795505 10.1111/j.1749-6632.2001.tb05644.x

[bib51] Oligomeric Proanthocyanidins (OPC). Monograph. Altern Med Rev 2003; 8: 442–450. 14653771

[bib52] Liu X, Zhou H-J, Rohdewald P. French maritime pine bark extract Pycnogenol dose-dependently lowers glucose in type 2 diabetic patients. Diabetes Care 2004; 27: 839. 10.2337/diacare.27.3.839 14988316

[bib53] Bester J, Buys AV, Lipinski B, Kell DB, Pretorius E. High ferritin levels have major effects on the morphology of erythrocytes in Alzheimer’s disease. Front Aging Neurosci 2013; 5: 88. 24367334 10.3389/fnagi.2013.00088 PMC3853801

[bib54] Dufrêne YF, Martínez-Martín D, Medalsy I, Alsteens D, Müller DJ. Multiparametric imaging of biological systems by force-distance curve-based AFM. Nat Methods 2013; 10: 847–854. 23985731 10.1038/nmeth.2602

[bib55] Berquand A. Quantitative imaging of living biological samples by PeakForce QNM atomic force microscopy. Bruker Appl Note 2011; 135: 1–10.

[bib56] Kolar P, Tomankova K, Malohlava J, Zapletalova J, Vujtek M, Safarova K et al. The effect of photodynamic treatment on the morphological and mechanical properties of the HeLa cell line. Gen Physiol Biophys 2013; 32: 337–346. 23817636 10.4149/gpb_2013042

[bib57] Sokolov I, Dokukin ME, Guz NV. Method for quantitative measurements of the elastic modulus of biological cells in AFM indentation experiments. Methods 2013; 60: 202–213. 23639869 10.1016/j.ymeth.2013.03.037

[bib58] Zhou Z, Zheng C, Li S, Zhou X, Liu Z, He Q et al. AFM nanoindentation detection of the elastic modulus of tongue squamous carcinoma cells with different metastatic potentials. Nanomedicine 2013; 9: 864–874. 23579203 10.1016/j.nano.2013.04.001

[bib59] Derjaguin BV, Muller VM, Toporov YP. Effect of contact deformations on the adhesion of particles. J Colloid Interface Sci 1975; 53: 314–326.

[bib60] Rosner B. Fundamentals of Biostatistics. 8th edn. Brooks Cole: Boston, 2015.

[bib61] Buys AV, Van Rooy MJ, Soma P, Van Papendorp D, Lipinski B, Pretorius E. Changes in red blood cell membrane structure in type 2 diabetes: a scanning electron and atomic force microscopy study. Cardiovasc Diabetol 2013; 12: 25. 23356738 10.1186/1475-2840-12-25 PMC3599682

[bib62] Anderson TW, Darling DA. A test of goodness of fit. J Am Stat Assoc 1954; 49: 765–769.

[bib63] Shapiro SS, Wilk MB. An analysis of variance test for normality (complete samples). Biometrika 1965; 52: 591–611.

[bib64] Wilcoxon F. Individual comparisons by ranking methods. Biometrics Bull 1945; 2: 1–8. 18903631

[bib65] Wilcox R. Comparing the variances of two dependent variables. J Stat Dist Appl 2015; 2: 1.

[bib66] Morgan W. A test for the significance of the difference between the two variances in a sample from a normal bivariate population. Biometrika 1939; 31: 13–19.

[bib67] Pitman E. A note on normal correlation. Biometrika 1939; 31: 9–12.

[bib68] Föller M, Huber SM, Lang F. Erythrocyte programmed cell death. IUBMB Life 2008; 60: 661–668. 18720418 10.1002/iub.106

[bib69] Lang E, Lang F. Mechanisms and pathophysiological significance of eryptosis, the suicidal erythrocyte death. Semin Cell Dev Biol 2015; 39: 35–42. 25636585 10.1016/j.semcdb.2015.01.009

[bib70] Lang E, Qadri SM, Lang F. Killing me softly - suicidal erythrocyte death. Int J Biochem Cell Biol 2012; 44: 1236–1243. 22561748 10.1016/j.biocel.2012.04.019

[bib71] Lang F, Jilani K, Lang E. Therapeutic potential of manipulating suicidal erythrocyte death. Expert Opin Ther Targets 2015; 19: 1219–1227. 26013571 10.1517/14728222.2015.1051306

[bib72] Lang F, Lang E, Föller M. Physiology and pathophysiology of eryptosis. Transfus Med Hemother 2012; 39: 308–314. 23801921 10.1159/000342534 PMC3678267

[bib73] Lang E, Lang F. Triggers, inhibitors, mechanisms, and significance of eryptosis: the suicidal erythrocyte death. Biomed Res Int 2015; 2015: 513518. 25821808 10.1155/2015/513518 PMC4364016

[bib74] Lang E, Zelenak C, Eberhard M, Bissinger R, Rotte A, Ghashghaeinia M et al. Impact of cyclin-dependent kinase CDK4 inhibition on eryptosis. Cell Physiol Biochem 2015; 37: 1178–1186. 26418250 10.1159/000430241

[bib75] Lang E, Jilani K, Bissinger R, Rexhepaj R, Zelenak C, Lupescu A et al. Vitamin D-rich diet in mice modulates erythrocyte survival. Kidney Blood Press Res 2015; 40: 403–412. 26227001 10.1159/000368517

[bib76] Lupescu A, Bissinger R, Goebel T, Salker MS, Alzoubi K, Liu G et al. Enhanced suicidal erythrocyte death contributing to anemia in the elderly. Cell Physiol Biochem 2015; 36: 773–783. 26021265 10.1159/000430137

[bib77] Qadri SM, Donkor DA, Bhakta V, Eltringham-Smith LJ, Dwivedi DJ, Moore JC et al. Phosphatidylserine externalization and procoagulant activation of erythrocytes induced by Pseudomonas aeruginosa virulence factor pyocyanin. J Cell Mol Med 2016; 20: 710–720. 26781477 10.1111/jcmm.12778 PMC5125577

[bib78] Ran Q, Xiang Y, Liu Y, Xiang L, Li F, Deng X et al. Eryptosis indices as a novel predictive parameter for biocompatibility of Fe3O4 magnetic nanoparticles on erythrocytes. Sci Rep 2015; 5: 16209. 26537855 10.1038/srep16209 PMC4633654

[bib79] Strydom MA, Bester J, Mbotwe S, Pretorius E. The effect of physiological levels of South African puff adder (Bitis arietans) snake venom on blood cells: an *in vitro* model. Sci Rep 2016; 6: 35988. 27775063 10.1038/srep35988 PMC5075924

[bib80] Bester J, Pretorius E. Effects of IL-1β, IL-6 and IL-8 on erythrocytes, platelets and clot viscoelasticity. Sci Rep 2016; 6: 32188. 27561337 10.1038/srep32188 PMC4999875

[bib81] Pretorius E. The adaptability of red blood cells. Cardiovasc Diabetol 2013; 12: 63. 23578325 10.1186/1475-2840-12-63 PMC3637111

[bib82] Pretorius E, Bester J. Viscoelasticity as a measurement of clot structure in poorly controlled type 2 diabetes patients: towards a precision and personalized medicine approach. Oncotarget 2016; 7: 50895–50907. 27447972 10.18632/oncotarget.10618 PMC5239445

[bib83] Bissinger R, Artunc F, Qadri SM, Lang F. Reduced erythrocyte survival in uremic patients under hemodialysis or Peritoneal dialysis. Kidney Blood Press Res 2016; 41: 966–977. 27941327 10.1159/000452600

[bib84] Bester J, Soma P, Kell DB, Pretorius E. Viscoelastic and ultrastructural characteristics of whole blood and plasma in Alzheimer-type dementia, and the possible role of bacterial lipopolysaccharides (LPS). Oncotarget 2015; 6: 35284–35303. 26462180 10.18632/oncotarget.6074 PMC4742105

[bib85] Potgieter M, Bester J, Kell DB, Pretorius E. The dormant blood microbiome in chronic, inflammatory diseases. FEMS Microbiol Rev 2015; 39: 567–591. 25940667 10.1093/femsre/fuv013 PMC4487407

[bib86] Pretorius E, Swanepoel AC, Buys AV, Vermeulen N, Duim W, Kell DB. Eryptosis as a marker of Parkinson’s disease. Aging 2014; 6: 788–819. 25411230 10.18632/aging.100695 PMC4247384

[bib87] Pretorius E, Bester J, Vermeulen N, Lipinski B, Gericke GS, Kell DB. Profound morphological changes in the erythrocytes and fibrin networks of patients with hemochromatosis or with hyperferritinemia, and their normalization by iron chelators and other agents. PLoS One 2014; 9: e85271. 24416376 10.1371/journal.pone.0085271 PMC3887013

[bib88] Packer L, Rimbach G, Virgili F. Antioxidant activity and biologic properties of a procyanidin-rich extract from pine (Pinus maritima) bark, pycnogenol. Free Radic Biol Med 1999; 27: 704–724. 10490291 10.1016/s0891-5849(99)00090-8

[bib89] Bors W, Michel C, Stettmaier K. Electron paramagnetic resonance studies of radical species of proanthocyanidins and gallate esters. Arch Biochem Biophys 2000; 374: 347–355. 10666317 10.1006/abbi.1999.1606

[bib90] Liu X, Wei J, Tan F, Zhou S, Würthwein G, Rohdewald P. Pycnogenol, French maritime pine bark extract, improves endothelial function of hypertensive patients. Life Sci 2004; 74: 855–862. 14659974 10.1016/j.lfs.2003.07.037

[bib91] Devaraj S, Vega-López S, Kaul N, Schönlau F, Rohdewald P, Jialal I. Supplementation with a pine bark extract rich in polyphenols increases plasma antioxidant capacity and alters the plasma lipoprotein profile. Lipids 2002; 37: 931–934. 12530550 10.1007/s11745-006-0982-3

[bib92] Mohanty J, Nagababu E, Rifkind JM. Red blood cell oxidative stress impairs oxygen delivery and induces red blood cell aging. Front Physiol 2014; 5: 84. 24616707 10.3389/fphys.2014.00084 PMC3937982

